# Correction: From space to time: Spatial inhomogeneities lead to the emergence of spatiotemporal sequences in spiking neuronal networks

**DOI:** 10.1371/journal.pcbi.1013637

**Published:** 2025-10-31

**Authors:** Sebastian Spreizer, Ad Aertsen, Arvind Kumar

An additional affiliation is missing for the third author. Arvind Kumar is also affiliated with the Science for Life Laboratory, Solna, Sweden.
