# Supplementary figures and images for: From space to time: Spatial inhomogeneities lead to the emergence of spatiotemporal sequences in spiking neuronal networks
Source: PLoS Comput Biol. 2019 Oct 25;15(10):e1007432. doi: 10.1371/journal.pcbi.1007432 (PMC6834288; doi:10.1371/journal.pcbi.1007432)

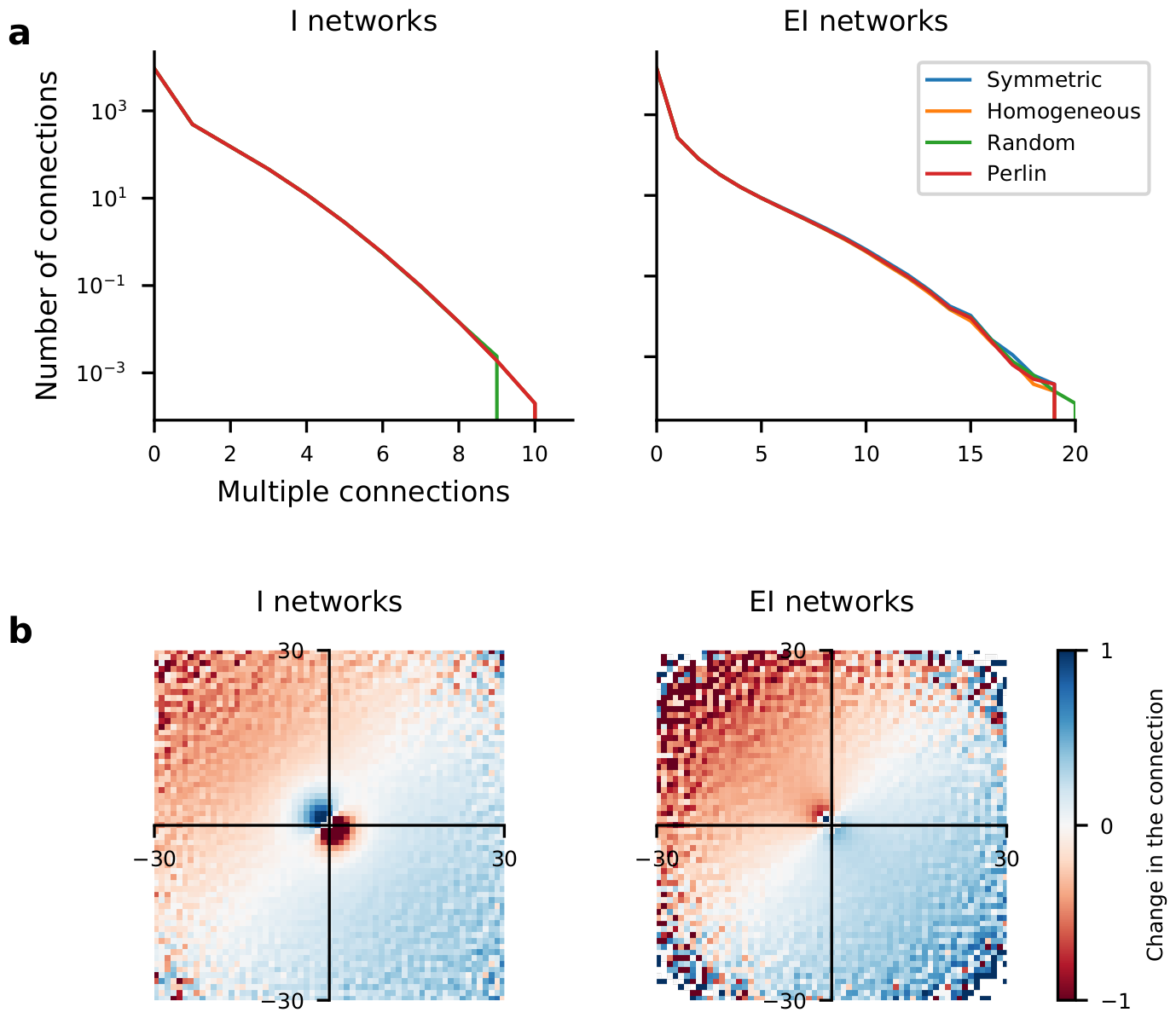

Supplement: S1 Fig — (a) Count distribution of multiple connections between any pair of neurons in an I-network (left) and in an EI-network (right). The multiple connections were formed primarily because of the connectivity rule (local connectivity). Note that the network configuration (as indicated by different colors of the curves) had only a minute influence on the distribution of multiple connections. (b:left) Average δconn for I-networks (average over all the neurons in the network). (b:right) Same as in the left panel, but for EI-networks. Forcing a neuron to make preferentially connections in the direction ϕ increased its connectivity in that direction: connectivity doubled in the immediate vicinity. Correspondingly, the connectivity was reduced by the same amount in the opposite direction. This change in the opposite direction is because we achieved asymmetry by shifting the connectivity cloud in the direction specified by ϕ (Fig 1). That is, in the immediate vicinity, the connection probability was doubled in the direction ϕ. This increase may look very large, but it nevertheless was not large enough to alter the probability of multiple connections in the network (a). Note that there is connectivity increase and corresponding decrease at larger distances, but such change was not of much consequence because at these large distances the connection probability was very small to begin with. (TIFF) [file pcbi.1007432.s001.tiff]

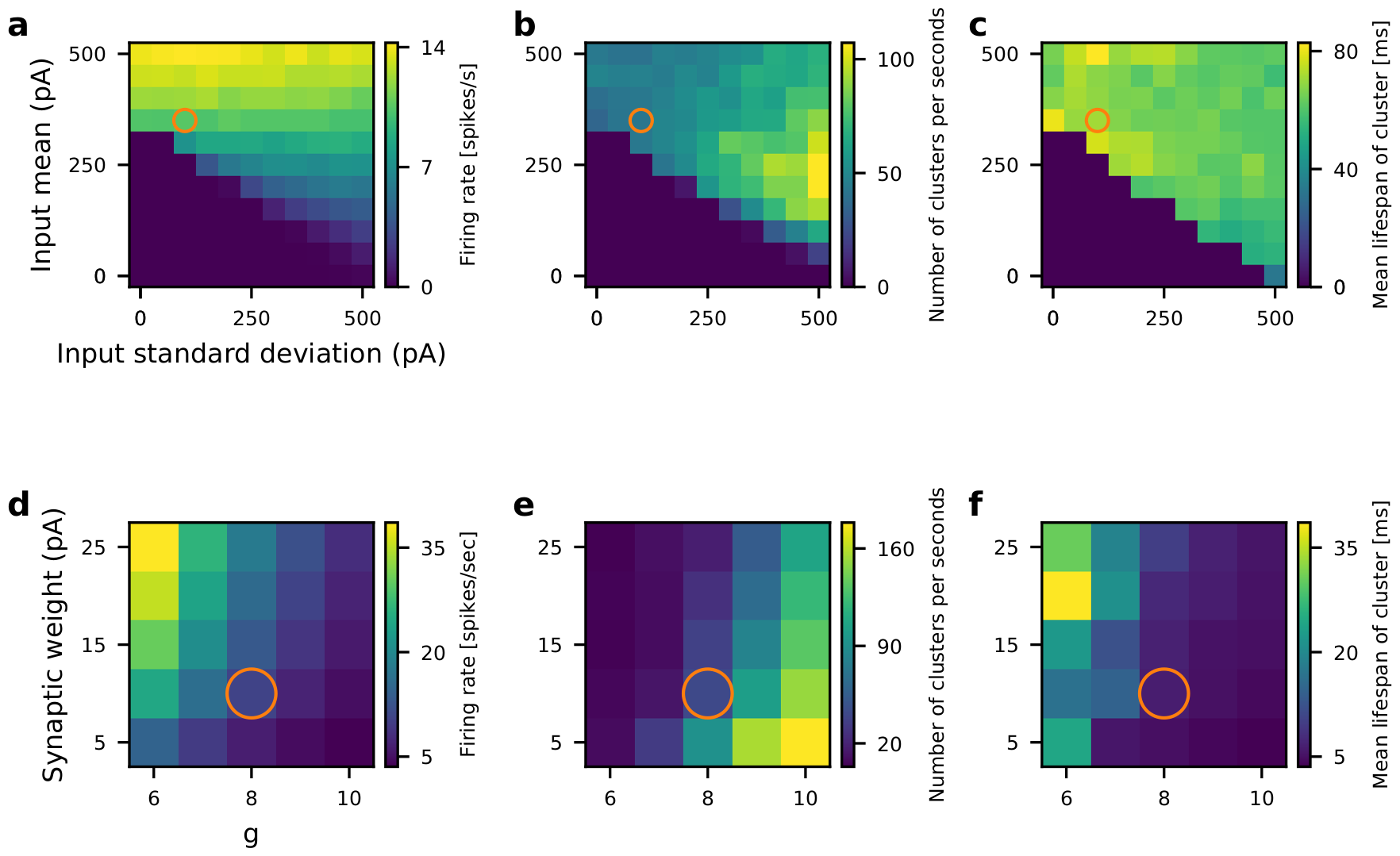

Supplement: S2 Fig — (a) Average firing rate of excitatory neurons as a function of the mean (ordinate) and standard deviation (abscissa) of the input noise to the all neurons. (b) The probability of observing an evoked STAS (cluster) as a function of the mean (ordinate) and standard deviation (abscissa) of the input noise to the all neurons. (c) The life span of an evoked STAS as a function of the mean (ordinate) and standard deviation (abscissa) of the input noise to the all neurons. The values for the excitation-inhibition balance used for panels a-c are marked by the orange circle in panels d-f. (d) Average firing rate of excitatory neurons as a function of excitatory synaptic weight (ordinate) and the ratio of recurrent inhibition and excitation (g, abscissa). (b) The probability of observing an evoked STAS as a function of excitatory synaptic weight (ordinate) and g (abscissa). (c) The life span of an evoked STAS as a function of excitatory synaptic weight (ordinate) and g (abscissa). The values of input mean and standard deviation used for panels d-f are marked by the orange circle in panels a-c. (TIFF) [file pcbi.1007432.s002.tiff]

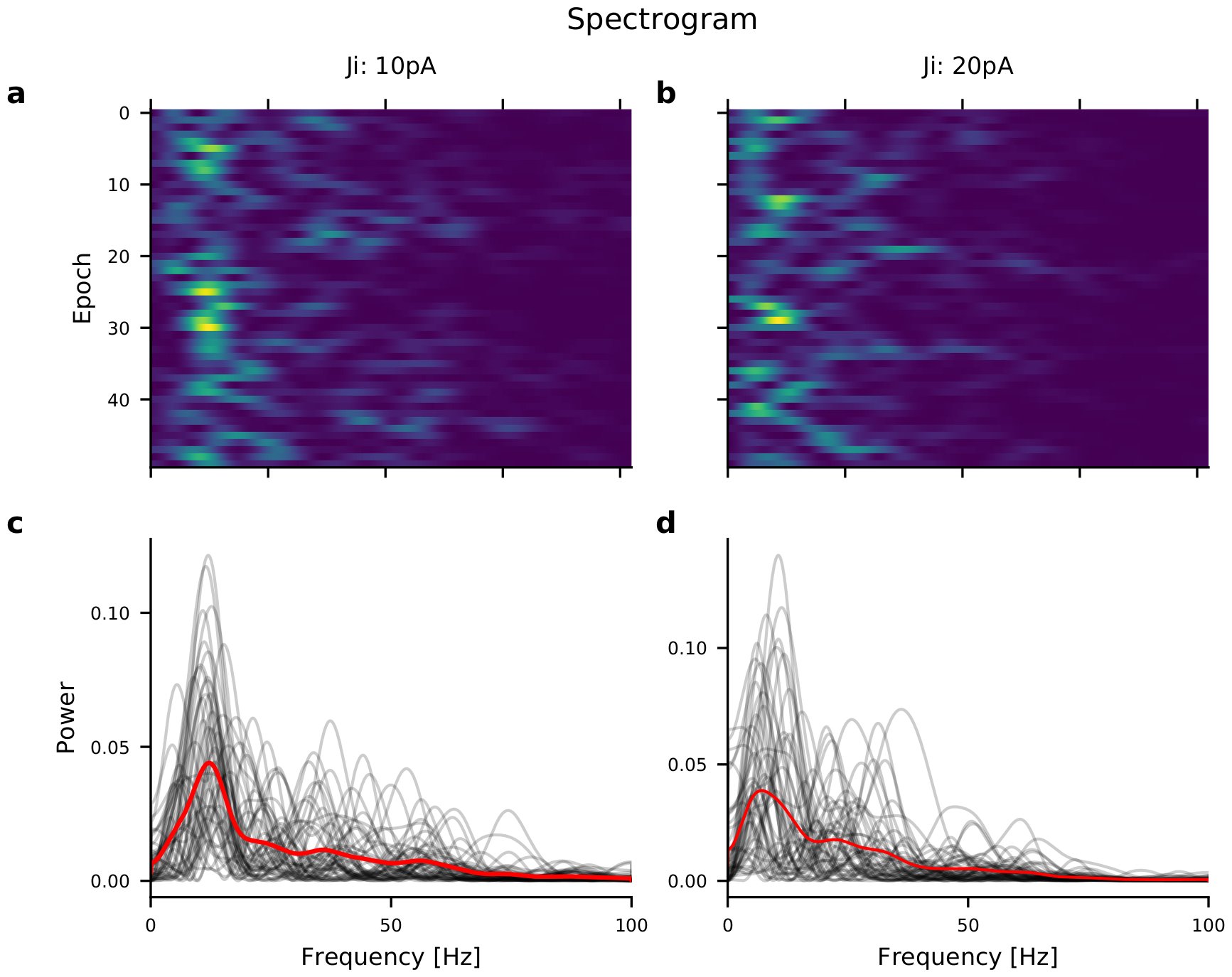

Supplement: S3 Fig — (a) Spectrogram for the EI-network with weaker recurrent synaptic strength (Ji = 10pA). The spectrogram was estimated by splitting the time series of population activity (bin width 5 ms) in 200 ms windows. Consecutive epochs overlapped for 150 ms duration. (c) Power in individual epochs (black curves) and the mean power (red curve) corresponding to the spectrogram shown in panel a as a function of frequency. (b) Same as in panel a for stronger recurrent inhibitory synaptic strength (Ji = 20 pA). (d) Same as in panel c but for the spectrogram shown in panel b. (TIFF) [file pcbi.1007432.s003.tiff]
