# Supplementary material for: From space to time: Spatial inhomogeneities lead to the emergence of spatiotemporal sequences in spiking neuronal networks
Source: PLoS Comput Biol. 2019 Oct 25;15(10):e1007432. doi: 10.1371/journal.pcbi.1007432 (PMC6834288; doi:10.1371/journal.pcbi.1007432)
Supplement: Attachment — Submitted filename: PointByPointReply.pdf [file pcbi.1007432.s005.pdf]

Dear Prof. Doiron and Prof. Marinazzo,

We thank the reviewers for their positive assessment of our work and their useful feedback. We have revised the manuscript as per their suggestions. In particular we have ensured that the methods are provided in sufficient detail to reproduce our results, even without the simulation code (which will be made available on github). Also, we have extended the text on the relevance of our findings. The new additions are marked in blue in the revised manuscript.

In the following we provide a point-by-point reply to the reviewers and specify how we have addressed each of their concerns.

Sincerely,
Arvind Kumar on the behalf of authors

Reviewer #1: The authors consider the question of how recurrent spiking neural network models can generate spatiotemporal sequences of activity, such as those believed to underly some neural comptuations. Through simulations and some intuition from the eigenvalue spectrum of their networks, they find that these sequences tend to be reliably generated when network connectivity satisfies a few reasonable and intuitive conditions. Notably, they included locally correlated anisotropies in the connectivity structure, which has not been considered in previous work to my knowledge, and which seems to be a key to generating coherent spatiotemporal sequences.

They additionally looked at the interaction between stimuli and these activity sequences, and proposed a mechanism through which neuromodulators might endow a network with the connectivity properties necessary for their generation. The study is interesting, novel, and potentially important for understanding dynamical computations in biologically plausible spiking networks.

The manuscript is suitable for publication in PLoS Comp Bio without any further changes.

We thank the reviewer for appreciating the importance, novelty and relevance of our work.

Reviewer #2: This manuscript develops a model of the potential mechanisms underlying the spatiotemporal patterns of neural activity observed in vivo. The focus is on neural sequences in the form of a sequential activation of neurons (spatiotemporal activity sequences-STAS). The authors propose that these patterns emerge as a result of shared asymmetries in the direction of axonal projections within cortical networks, specifically, neighbouring neurons may have a bias to project in a random but shared direction. Thus creating preferred channels by which activity will flow. The results seem solid, and to strongly support the conclusions, furthermore the model seems fairly systematic. The paper certainly adds to our understanding of the potential influence of shared axonal projection anisotropies in cortical networks, my main concern however, in the context of the current journal, revolves around biological relevance.

We thank the reviewer for the appreciation and constructive comments.

The mechanisms underlying sequence generation is an important one, and the authors cite many experimental papers that have observed these patterns (ref. 3-11). A potential concern is that to the best of my knowledge none of these papers suggest that there is any spatial organization to these observed patterns, indeed as I understand it in most cases it is explicitly not the case that neurons that fire closer together in time are located close together in space—this certainly does not seem to be the case in the birdsong system as I believe recent work by Michael Long suggests. It is stated that “the key prediction” (li 474) of the models is that brain regions generating STAS, should have asymmetric network connectivity. It seems that first and foremost that the key prediction/assumption is that STASs are spatially organized, and while I may be mistaken, to the best of my knowledge this assumption is not consistent with the data.

We agree with the reviewer that at the level of spiking activity of individual neurons, so far there is no direct evidence (to the best of our knowledge) of spatial organization, even though a number of papers have shown that neighboring neurons are more correlated than those far away (e.g. Peyrache et al. 2012 PNAS (during sleep); Minderer et al. 2019 Neuron (in awake behaving animals)). However, when we observe the activity of many neurons e.g. by voltage sensitive dye imaging or local field potentials, spatially organized waves of neuronal activity are clearly visible (e.g. see review by Muller et al. 2018 and references therein). Hence, we think it is reasonable to argue that the lack of evidence for spatial organization of sequences of neuronal activity results from sparse sampling of neurons by extracellular electrodes or from poor temporal resolution of calcium imaging.

An implication of structure in connectivity emerging in our model is that neurons should have correlation in their in-degree and out-degree. This point was not included in the original manuscript but we have that now highlighted in the revised version [Lines 312-316]. With this point in mind, even if sequences in the brain are not spatially organized, our model provides the necessary conditions to generate sequences intrinsically, i.e. their correlation between in-degree and out-degrees of the neurons in the network. We have extended the discussion to include these points. [see Lines 415-428; Lines 506-510]

Figure 4. It is stated that both I and EI networks exhibited clear oscillations in all configurations. I was not able to clearly see these oscillations in the power spectra of Figure 4, particularly for the EI random networks. In many cases the peaks were not particularly obvious, or in the range of 30-60 Hz, or was it clear that they were significant.

We agree with the reviewer that oscillations are not prominent in the EI-networks. In fact, oscillations when existing in the EI-networks were transient and of lower frequency than in the II-networks. We have revised this Figure and the corresponding text accordingly [see changes made in the section ‘Co-existence of activity sequences and network oscillations’; Lines 192-213]. In addition, we have now

added a new Supplementary Figure showing the transient nature of the oscillations in EI-networks [See Supplementary Figure S4].

Since the goal of the paper is to present a biologically plausible model I'm not sure the I-networks really contributed to the paper as I-networks are not biologically entities.

We respectfully disagree with the reviewer. Networks in the basal ganglia (i.e. striatum, globus pallidus) and amygdala (in particular the central amygdala) are purely inhibitory and play a crucial role in a variety of brain functions. In the striatum, experimental work clearly shows sequential activity (Bakhurin et al. J. Neurosci. 2017). Therefore, the part dealing with the I-networks is as important as the part dealing with the EI-networks. We realize that we did not motivate the choice of I-networks in the manuscript. In the revised version we have corrected this [see Lines 63-65; Lines 549-550; Lines 558-559].

It would be helpful to provide the equations for the synaptic currents.

We apologize for missing out on this. We have now provided the equations for synaptic currents and also ensured that methods are explicitly stated to facilitate the reproduction of the results. [Line 532-535]
